# Mid-Infrared Photothermal–Fluorescence In Situ Hybridization for Functional Analysis and Genetic Identification of Single Cells

**DOI:** 10.1021/acs.analchem.2c04474

**Published:** 2023-01-18

**Authors:** Yeran Bai, Zhongyue Guo, Fátima C. Pereira, Michael Wagner, Ji-Xin Cheng

**Affiliations:** † Department of Electrical and Computer Engineering, Boston University, Boston, Massachusetts 02215, United States; ‡ Department of Biomedical Engineering, Boston University, Boston, Massachusetts 02215, United States; § Photonics Center, Boston University, Boston, Massachusetts 02215, United States; ∥ Centre for Microbiology and Environmental Systems Science, Department of Microbiology and Ecosystem Science, University of Vienna, Vienna 1030, Austria; ⊥ Department of Chemistry and Bioscience, Aalborg University, Aalborg 9220, Denmark

## Abstract

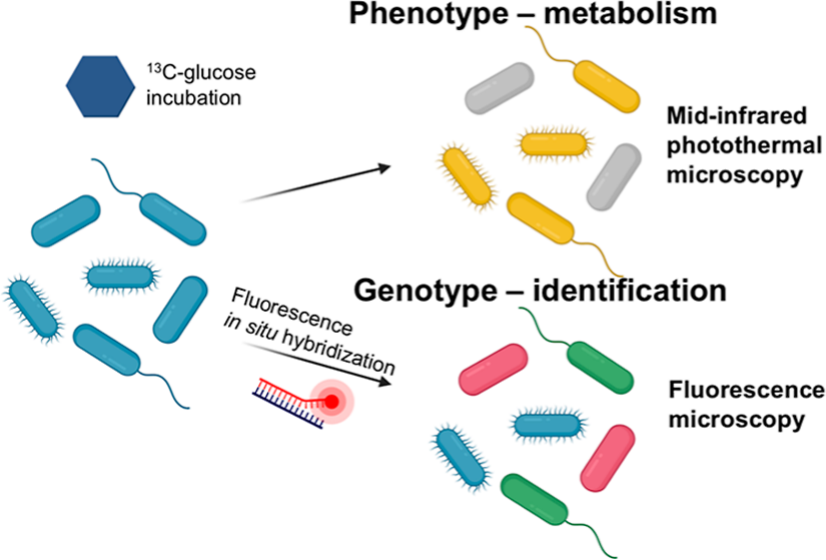

Simultaneous identification and metabolic analysis of microbes with single-cell resolution and high throughput are necessary to answer the question of “who eats what, when, and where” in complex microbial communities. Here, we present a mid-infrared photothermal–fluorescence in situ hybridization (MIP–FISH) platform that enables direct bridging of genotype and phenotype. Through multiple improvements of MIP imaging, the sensitive detection of isotopically labeled compounds incorporated into proteins of individual bacterial cells became possible, while simultaneous detection of FISH labeling with rRNA-targeted probes enabled the identification of the analyzed cells. In proof-of-concept experiments, we showed that the clear spectral red shift in the protein amide I region due to incorporation of ^13^C atoms originating from ^13^C-labeled glucose can be exploited by MIP–FISH to discriminate and identify ^13^C-labeled bacterial cells within a complex human gut microbiome sample. The presented methods open new opportunities for single-cell structure–function analyses for microbiology.

## Introduction

In eukaryotic cell biology, measuring single-cell behaviors and cell-to-cell heterogeneity in a complex environment is key to understanding cellular interactions in different physiological conditions.^1−7^ For microorganisms, the heterogeneity in genotypic and phenotypic traits has a direct impact on human health and the functioning of environmental microbiomes.^8−11^ Consequently, the rapidly developing single-cell technologies have revolutionized microbiology.^12−16^ Among omics-based analyses, single-cell metabolomics provides the most immediate and dynamic picture of the functionality of a cell, but it is arguably the most difficult to measure.^17,18^ Due to the small amount of metabolites present in single cells and the inability for amplification, detection sensitivity challenges are posed on metabolomics technology, especially when analyzing the comparably small bacterial and archaeal cells. Additionally, as the function of a cell in a given set of physiochemical conditions is a variable and dynamic property that cannot be reliably predicted from either metabolic reconstructions or genomics data alone,^12^ genotyping integrated with metabolic analysis provides a better way to understand how microorganisms interact with their biotic and abiotic environment. Therefore, technologies that help bridge genotype and phenotype of microbes at the single-cell level are in high demand.^19−22^

Vibrational spectroscopy with stable isotope probing has recently emerged as a novel platform for single-cell metabolism profiling.^23−30^ Compared to mass spectrometry, vibrational spectroscopy is nondestructive and promises the compatibility with genotypic analysis.^17^ For stable isotope probing, cells are either incubated with specific substrates carrying isotopically labeled atoms (most commonly ^13^C, ^15^N, ^18^O, and ^2^H) or with compounds such as heavy water (^2^H_2_O/D_2_O) that are incorporated by all metabolic active cells and thus serve as general activity markers.^28^ The newly anabolized biomolecules including lipids, proteins, and nucleic acids that contain the substrate-derived isotopes can be detected with single-cell resolution by investigating the red-shifted vibrational peaks due to the isotopic effect. Raman spectroscopy has been successfully applied to study bacterial metabolic activities by tracking incorporation of ^2^H (deuterium) from D_2_O or ^13^C from ^13^C-labeled substrates into single bacterial cell biomass.^21,31^ In these studies, the isotope-labeled cells were simultaneously identified using fluorescence in situ hybridization (FISH) with rRNA-targeted oligonucleotide probes. However, a spontaneous Raman spectrum from a single bacterium takes about 20 s to acquire, resulting in limited throughput that prevents large-scale analysis. Additionally, Raman spectroscopy is sometimes challenging to integrate with fluorescence-based genotyping methods because Raman scattering and fluorescence emission can result in spectral overlap, which then complicates spectral interpretation.^31^ Recently, we reported on the combination of stimulated Raman scattering and FISH (SRS–FISH) that greatly boosted the Raman spectral acquisition speed and enabled an increase in throughput of analyzed microbial cells by 2–3 orders of magnitude.^20^ In this study, the activities of selected human gut microbiome members after incubation with different mucosal sugars in the presence of heavy water were investigated at high throughput. However, the direct visualization of sugar metabolism by tracking the incorporation of the ^13^C-labeled substrates by microbiome members has not yet been achieved with SRS–FISH. Additionally, SRS imaging required that the analyzed bacteria were immersed in liquid, while the two-photon fluorescence imaging used for detection of FISH-labeled cells turned out to be more efficient in dry samples to avoid photobleaching, which complicated the experimental procedures.^20^ In contrast, infrared (IR) absorption can be applied to study cell metabolism^32^ while not suffering from fluorescence background. It should also not require different sample conditions for optimal IR and fluorescence measurements. However, the spatial resolution of conventional IR microscopy is limited to several micrometers,^33,34^ which hinders imaging of individual bacteria and co-recording of IR spectra and genotypic-informative FISH images.

The recently developed mid-IR photothermal (MIP) imaging addresses these limitations.^35−41^ In MIP, two lasers in the mid-IR and the visible regions are used. When the modulated mid-IR light is absorbed by the sample, it leads to sample heating and expansion (photothermal effect). The visible beam passing through the sample redirects its propagation direction due to the photothermal effect. A far-field photosensor detects the periodically modulated probe photons, and an image is created through pixel-by-pixel scanning or in a widefield manner. MIP has been successfully applied to image a range of organisms from a whole nematode *Caenorhabditis elegans* to a single virus at sub-micrometer spatial resolution.^38,39,42−47^ The spectra from single bacteria have been recorded with MIP with high spectral fidelity and 290 nm spatial resolution.^48,49^ Additionally, the MIP signal could be detected from fluorescence intensity fluctuation for fluorophore-loaded samples.^50,51^ However, so far, there is no demonstration of MIP for simultaneous bacterial FISH-genotyping and metabolic imaging *via* isotope probing.

Here, we present a MIP–FISH platform that enables high-throughput metabolic imaging and identification of bacteria with single-cell resolution. By using oligonucleotide probes tagged with fluorophores to target signature regions in ribosomal RNA (rRNA) genes, FISH has become an indispensable tool for rapid and direct single-cell identification of microbes.^52^ In this work, we greatly improved the performance of a widefield MIP microscope through multiple optimizations such as the utilization of a nanosecond laser as the probe source. We then incorporated a fluorescence module on the widefield MIP to enable a coregistered MIP and fluorescence imaging from the same cells. To demonstrate the high-throughput metabolic imaging capability of MIP–FISH, we imaged the newly synthesized protein in hundreds of *Escherichia coli* cells from ^13^C-labeled glucose in seconds, with single-cell resolution. Simultaneous identification of bacterial taxa and metabolism profiling was demonstrated by imaging bacterial mixtures including a spiked gut microbiome sample. Collectively, our results demonstrate the capability of high-throughput microbial phenotyping of metabolism and genotyping with single-cell resolution through MIP–FISH.

## Experimental Section

### MIP–FISH Microscope

**Figure 1:**
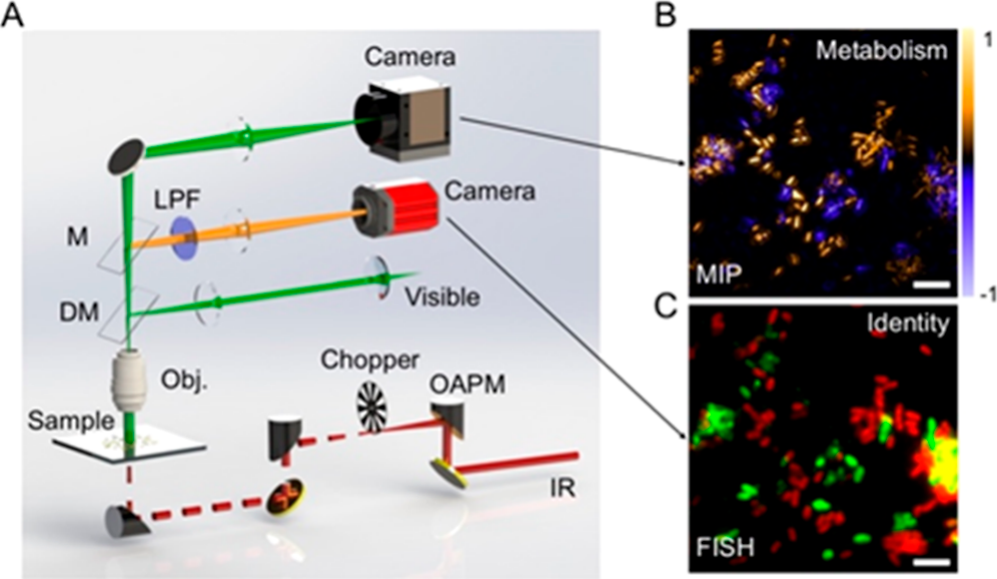
Schematic of MIP–FISH for in situ bacteria identification and phenotypical metabolic imaging. (A) The setup was based on a widefield MIP with the incorporation of a fluorescence module. OAPM, off-axis parabolic mirror. DM, dichroic mirror. LPF, long pass filter. M, mirror. (B) The subtraction image of MIP signals at two IR wavenumbers provides information on cellular metabolism. Positive values (in yellow) indicate active incorporation of ^13^C from labeled substrates (here ^13^C-glucose) into protein, while negative values (in blue) indicate no ^13^C incorporation. (C) The fluorescence image detects a signal from FISH with rRNA-targeted probes, enabling the identification of bacterial taxa (*Escherichia coli* in red and *Bacteroides thetaiotaomicron* in green). Scale bars: 5 μm.

Figure 1A shows a schematic illustration of the MIP–FISH microscope. For MIP imaging, the mid-IR pump source is an optical parametric oscillator (OPO) laser (Firefly-LW, M Squared Lasers) with 20 ns pulse duration and 20 kHz repetition rate. The visible probe is a nanosecond laser (NPL52C, Thorlabs) with a center wavelength of 520 nm and a pulse width of 129 ns. The mid-IR beam was modulated using an optical chopper (MC2000B, Thorlabs) with a duty cycle of 50%. The mid-IR was optically chopped into pulse trains with a modulation frequency of 635 Hz, and around 16 bursts of IR pulses are in the period of one camera exposure time. The microscopy objective (MPLFLN Olympus, 100X, NA 0.9) was used to focus the visible light onto the sample as well as to collect the reflected light. To record the sample scattered light for MIP imaging, a high full-well-capacity camera (Q-2HFW, Adimec) was used. For FISH imaging, a fluorescence module composed of a fluorescence camera (CS235MU, Thorlabs), a dichroic beam splitter, and filter sets were installed on the MIP microscope. An additional continuous wave 638 nm laser (0638-06-01, cobalt) was aligned with a 520 nm laser for additional fluorophore excitation. For MIP imaging, the IR power before the microscope was 32.9 and 34.8 mW at 1612 and 1656 cm^–1^, respectively. All presented images were normalized with IR powers at corresponding wavelengths. The visible power was less than 1 mW before the microscope. Unless otherwise noted, the MIP images at one IR wavenumber were acquired at the speed of 2.4 s per image. For fluorescence imaging, the exposure time of the fluorescence camera was 1 s with a gain of 20 dB.

### *E. coli*^13^C-Glucose Isotope Labeling and Sample Preparation

**Figure 2:**
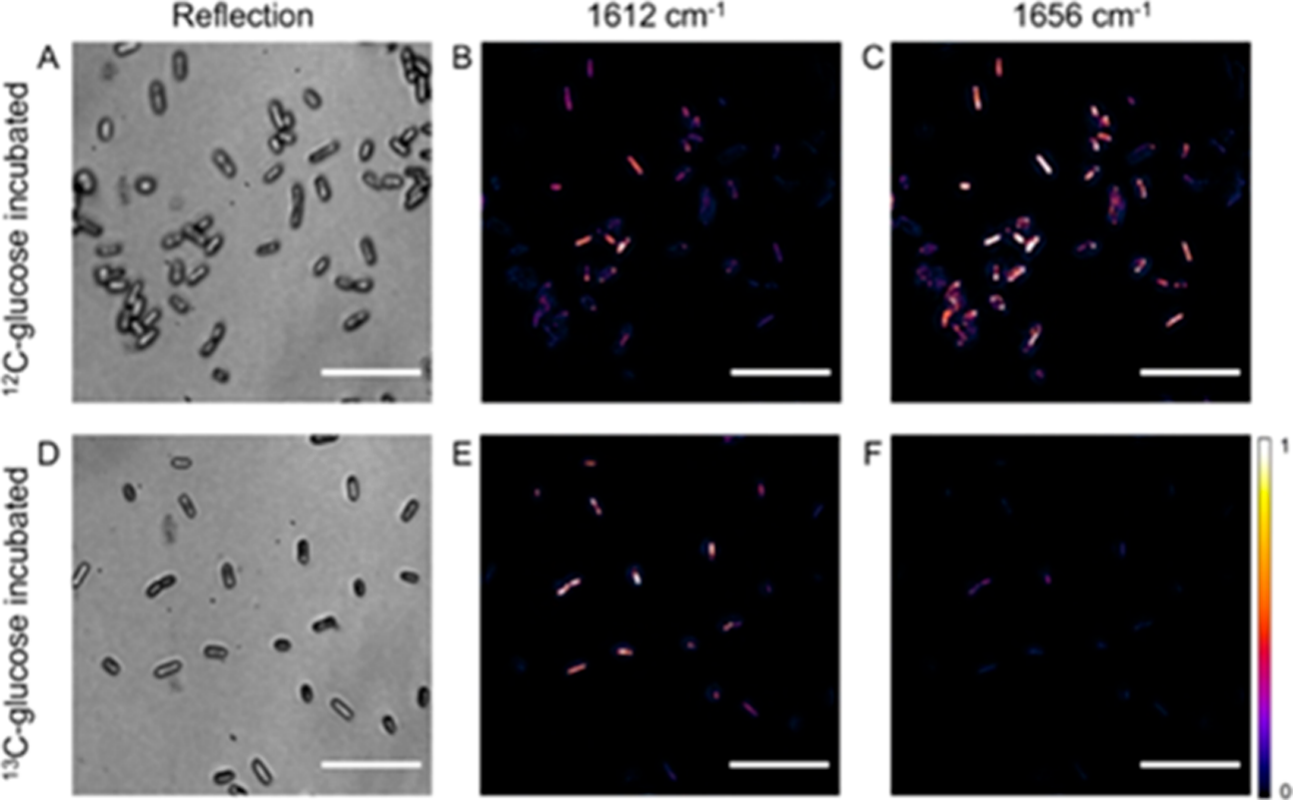
Single-cell metabolic imaging of ^13^C-glucose incorporation by widefield MIP. Reflection and MIP images at two key protein amide I wavenumbers (1612 and 1656 cm^–1^) for *E. coli* cells incubated with ^12^C-glucose (A–C) or ^13^C-glucose (D–F). Scale bars 10 μm.

**Figure 3:**
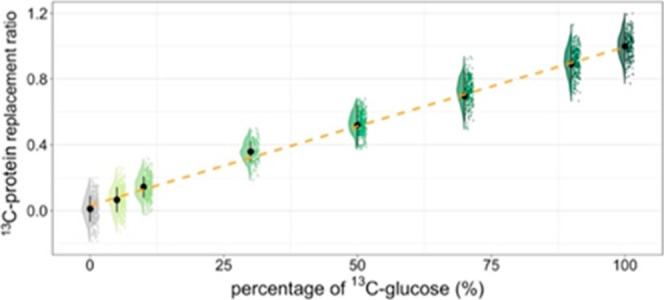
High detection sensitivity for ^13^C-incorporation. ^13^C-protein replacement ratio of *E. coli* cells grown for 24 h in minimal medium supplemented with 0.2% (w/v) of glucose at varying percentages of the total glucose in the form of ^13^C-glucose (0, 5, 10, 30, 50, 70, 90, and 100%). More than 155 cells in each group were measured to produce the mean and standard deviation. A significant difference was observed between the 0 and the 5% ^13^C-glucose-incubated cells (pairwise *t*-test, *p* = 3.86 × 10^–22^). A linear regression between the percentage of ^13^C-glucose and ^13^C-protein replacement ratio is shown as a dashed line (*R*^2^ = 0.9982).

For data presented in Figures 2 and 3, *E. coli* BW25113 was inoculated from a single colony and precultured in a nutrient-rich medium (either tryptic soy broth or Mueller Hinton broth) for 3 h to reach the log phase. The optical density at 600 nm was measured to estimate the concentration of cells per milliliter. Then, the cultures were diluted to a concentration around 5 × 10^5^ cfu/mL in a M9 minimal medium. The M9 minimal medium was supplemented with ^12^C-glucose or ^13^C-glucose, or a varying volume mixture of both, at a final concentration of 0.2% (w/v). The ^13^C-glucose used (d-glucose U–^13^C_6_, 99%, Cambridge Isotope Laboratories) was universally labeled—so that all carbon atoms were replaced with ^13^C atoms. Cells were harvested by centrifugation at 11,000 rpm and 4 °C for 3 min after 24 h of aerobic incubation at 37 °C with glucose. The bacterial cells were then fixed with 10% formalin at 4 °C overnight. Multiple rounds of centrifugation and washes with deionized water were performed to remove the remaining fixative. A 2 μL drop of the concentrated cell solution in water was deposited on a poly-l-lysine-coated IR-transparent silicon coverslip (silicon 2018, University Wafers) and dried in air.

### Multispecies Sample and Fluorescence In Situ Hybridization

**Figure 4:**
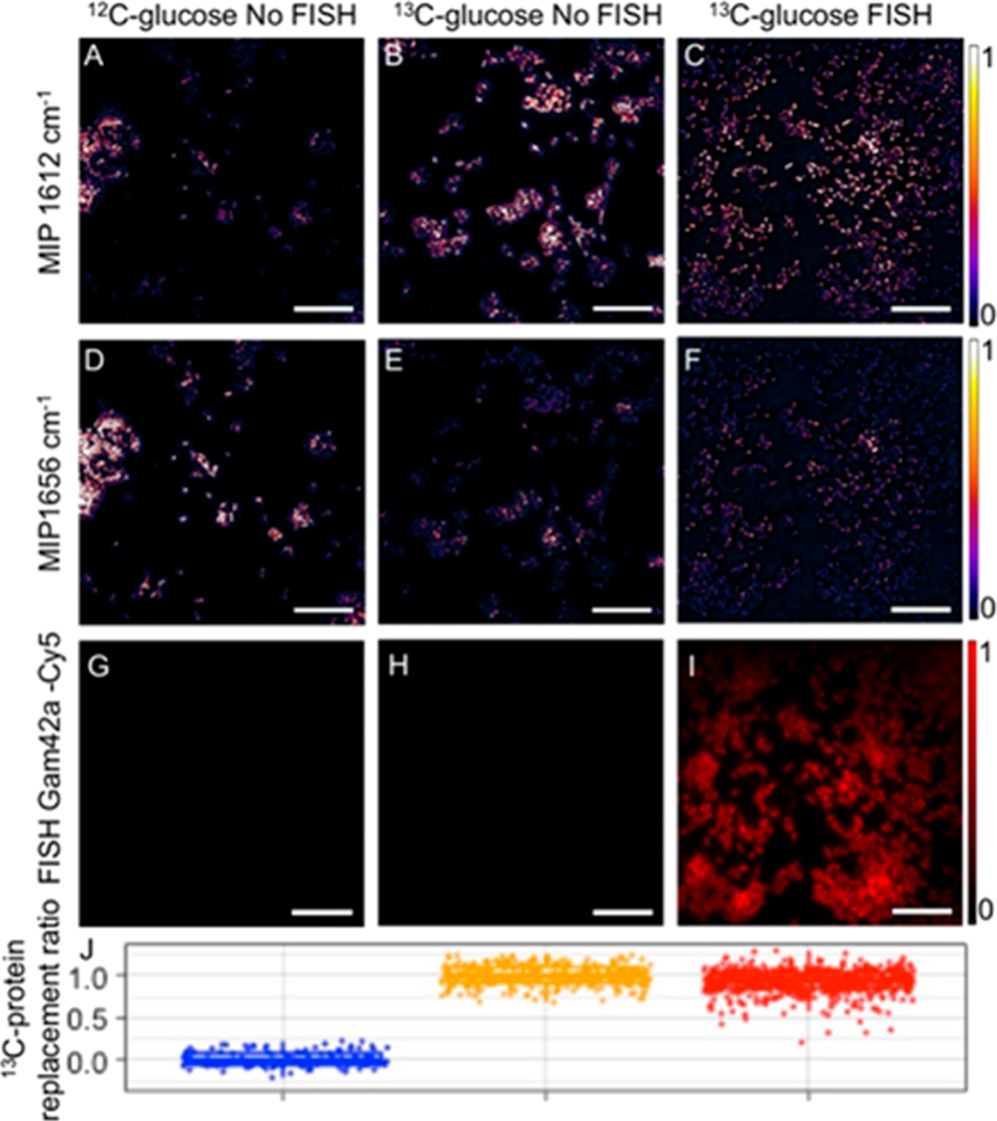
FISH is compatible with MIP metabolic imaging. *E. coli* cells grown in a ^12^C-glucose-containing medium with no FISH labeling were imaged at the two amide I peak wavenumbers as well as by recording fluorescence in the Cy5 channel (A,D,G). ^13^C-glucose incubated *E. coli* with and without FISH labeling was imaged at the same channels (B,E,H and C,F,I) using identical settings. (J) The ^13^C-protein replacement ratio was calculated for each incubation group. A slight reduction (7.5%) was observed for the ^13^C-protein replacement ratio between groups with and without FISH labeling. Scale bars 10 μm.

**Figure 5:**
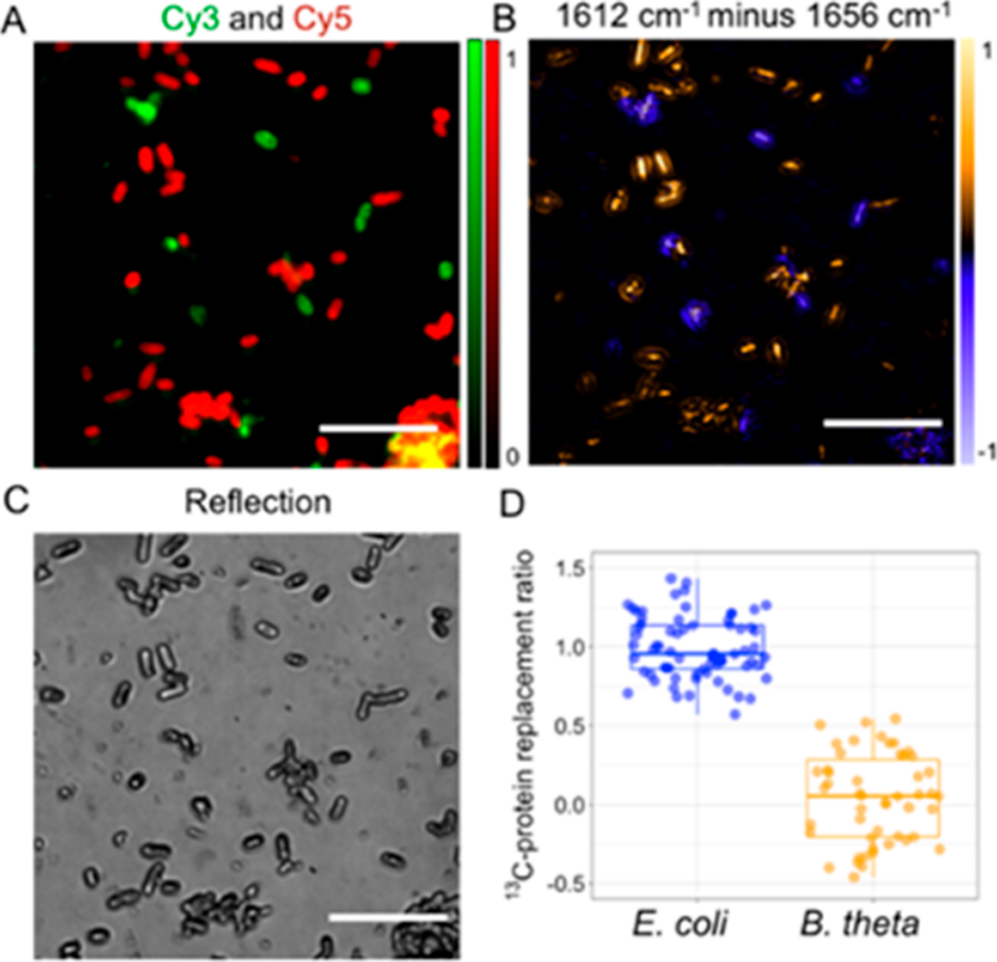
MIP–FISH imaging of bacterial mixtures. *E. coli* cells were incubated with 0.4% (w/v) ^13^C-glucose and hybridized with a Gam42a–Cy5 oligonucleotide probe, while *B. theta* cells grown in the presence of ^12^C-glucose were hybridized with a Bac303–Cy3 oligonucleotide probe. (A) Fluorescence imaging for identification of *E. coli* (red) and *B. theta* (green). Scale bars 10 μm. (B) Subtraction of two MIP images (intensity at 1612 cm^–1^ minus intensity at 1656 cm^–1^) showed that a portion of the cells have incorporated ^13^C into the protein (in yellow), while other cells showed no ^13^C labeling (in blue). (C) Reflection image shows the cell morphology of the bacterial mixture. (D) Quantification of the ^13^C-protein replacement ratio. (Pairwise *t*-test: *p* = 9.74 × 10^–33^.)

**Figure 6:**
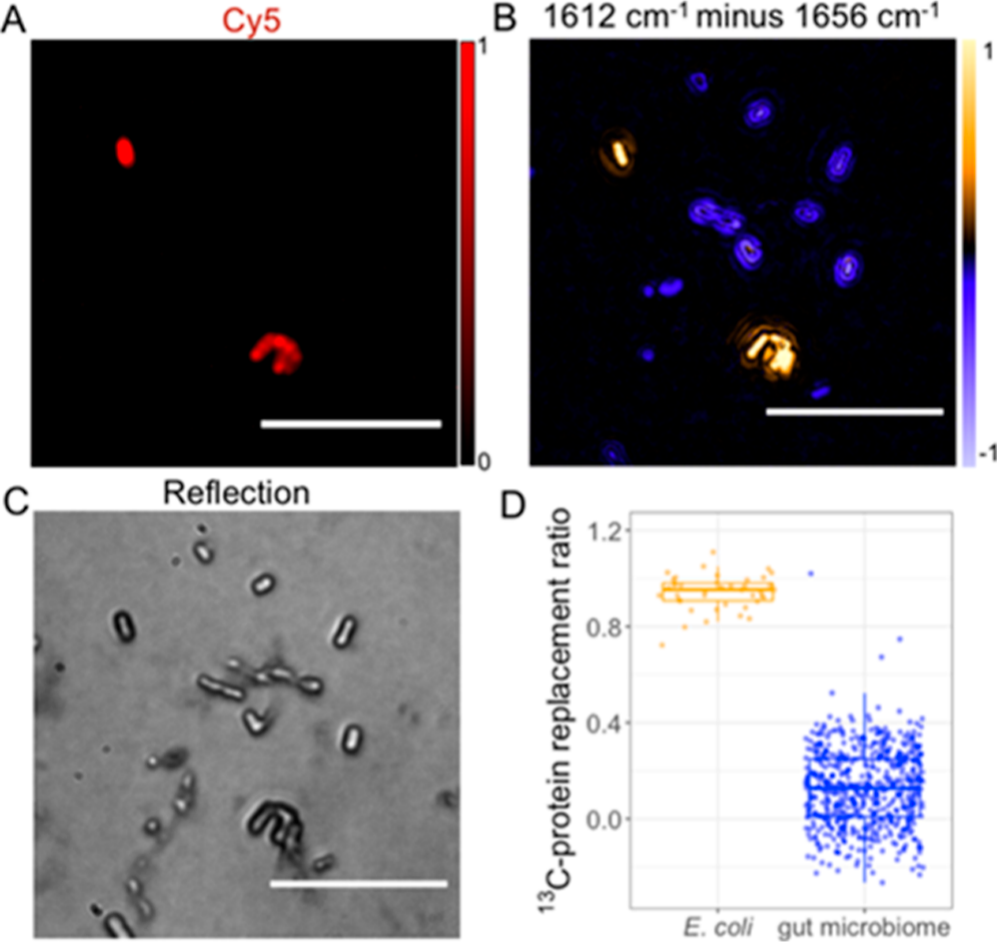
MIP–FISH imaging of a gut microbiome sample with spiked ^13^C-labeled *E. coli* cells. ^13^C-glucose fully labeled *E. coli* cells that were FISH labeled with Cy5 were mixed with an isotopically unlabeled human gut microbiome sample. (A) Fluorescence imaging enabled localization of the added *E. coli* cells in the complex sample. Scale bars 10 μm. (B) MIP subtraction image indicated ^13^C-labeling for *E. coli* (in yellow). (C) Reflection image of the sample mixture. (D) Quantification of the ^13^C-protein replacement ratio. (Pairwise *t*-test: *p* = 7.69 × 10^–24^.)

For data presented in Figures 4–6, *E. coli* K-12 (DSM 498) was grown aerobically at 37 °C in a M9 minimal medium containing 0.4% (w/v) of either ^12^C-glucose (unlabeled d-glucose, 99.5%, Sigma-Aldrich) or ^13^C-glucose (d-glucose–^13^C_6_, 99%, Sigma-Aldrich). Cells were grown overnight in a M9 medium containing unlabeled glucose and diluted 1:100 in 5 mL of fresh medium containing either ^12^C- or ^13^C -glucose. *Bacteroides thetaiotaomicron* (DSM 2079) (*Bacteroides theta*) cells were grown anaerobically (in a Coy Labs anaerobic chamber containing an atmosphere of 85% N_2_, 10% CO_2_, and 5% H_2_) in *Bacteroides* defined minimal medium (BMM) containing 0.5% (w/v) of either ^12^C-glucose (unlabeled d-glucose, 99.5%, Sigma-Aldrich) or ^13^C-glucose (d-glucose-^13^C_6_, 99%, Sigma-Aldrich).^53^ Cells were grown overnight in BMM containing unlabeled glucose and diluted 1:100 in 5 mL of fresh medium containing either ^12^C- or ^13^C-glucose. *E. coli* and *B. theta* cells were harvested by centrifugation at the late exponential phase (9 h of growth for *E. coli* and 12 h of growth for *B. theta*) and immediately fixed in 4% formaldehyde in phosphate-buffered saline (PBS) for 2 h at 4 °C. Cells were subsequently washed once with PBS, resuspended in 1 mL of a 50% (v/v) mixture of PBS and 96% ethanol, and finally stored at −20 °C until further use. *E. coli* cells were subsequently hybridized with the Gam42a probe tagged with the Cy5 fluorophore, and *B. theta* cells were hybridized with the Bac303 probe tagged with the Cy3 fluorophore following a standard FISH protocol (Supporting Information Methods and Table S1). Hybridized *E. coli* and *B. theta* cells were mixed, and 2 μL of this mixture was spotted on the poly-l-lysine-coated silicon coverslips and allowed to dry in air protected from light. In addition, a cultured human gut microbiome sample stored in PBS (Supporting Information Methods) and prehybridized *E. coli* cells that have been stored in PBS/ethanol were gently mixed in an Eppendorf tube and subsequently spotted on poly-l-lysine-coated silicon coverslips. Excess salt was removed by adding 2 μL of Milli-Q water onto the dried spot. Subsequently, the water was gently blown away, and the spot was dried again at room temperature protected from light.

### ^13^C-Protein Replacement Ratio Quantification

For high-throughput, single-cell analysis, the regions of interests were determined based on the reflection images. To quantify metabolic activity, we defined the ^13^C-protein replacement ratio as the relative contribution of the ^13^C-protein to the whole protein (Figure S1). The contribution was calculated based on four coefficients obtained from two reference samples at the original amide I (1656 cm^–1^) and the shifted amide I (1612 cm^–1^) bands. Since different culture and treatment conditions were used, we obtained different coefficients for in-group comparison. The details of coefficients and statistics for reported experiments are listed in Table S2. We also recorded the coefficients for the same reference sample on different days and observed less than 6% variation (Table S3).

## Results and Discussion

### MIP–FISH Platform

We improved the performance of the previously reported first-generation widefield MIP microscope^41^ and achieved an over 2 orders of magnitude increase of the imaging speed by making the following modifications: (1) a LED was used as the probe source in the first-generation MIP setup with a minimal pulse width of 900 ns, which was ideal for micron-sized polymer beads since the decay constant is around several microseconds.^41,54^ However, the signal produced from a single bacterium is much weaker than that of a polymer bead, and the decay constant is much shorter, reaching to 280 ns.^35^ Therefore, we coupled a nanosecond laser with a pulse width of 129 ns to match the decay constant to improve the detection sensitivity for bacteria. (2) MIP is a shot noise-limited technique, and the signal-to-noise ratio (SNR) is proportional to the total probe photon received.^41^ Therefore, we incorporated a high full-well-capacity (2 Me^–^) camera to the current setup. (3) To accommodate the high full-well-capacity camera with a pixel size of 12 μm, we used a high-magnification and high numerical aperture objective. Other improvements including shortening of the IR pathlength, galvo scanner adjusting the pointing of the IR beam when tuning wavenumbers, and adding a laser speckle reduction module synergistically worked together to push the MIP imaging speed. As a comparison, we previously achieved 2 frames/s for 1 μm polymethyl methacrylate (PMMA) beads imaging with a field of view of around 20 μm and a SNR of 24.^54^ By implementing the optimizations, we achieved 635 frames/s for 500 nm PMMA beads imaging with a field of view of around 60 μm and a SNR of 31 (Figure S3). Collectively, these optimizations were essential to adapt the technique for high-throughput bacterial metabolic phenotyping.

The MIP–FISH microscope is schematically shown in Figure 1A. Briefly, a fluorescence module was integrated into the MIP microscope sharing the visible illumination. The mid-IR pulses are modulated with an optical chopper to a burst of pules matching the camera frame rate. In this work, we focused on imaging of the fluorophores Cy3 and Cy5, which are widely used for FISH-based detection of microbes. The 520 nm nanosecond probe source also served as the Cy3 fluorophore excitation source. The Cy5 fluorophore was excited with a second visible beam with a center wavelength of 638 nm aligned with the 520 nm laser. Due to the different requirement of MIP and FISH imaging for cameras, we separated the two detection paths and used two cameras for recording the sample scattered light and the fluorophore-emitted light. For the bacteria taxa-specific fluorescence detection from FISH, we chose a camera with high quantum efficiency and low readout noise for the low photon-budget condition. The metabolic activity of the cells was characterized by two IR wavenumber MIP imaging (Figure 1B), while the identity of the cells was provided by two-color fluorescence imaging (Figure 1C).

### High-Throughput, High-Sensitivity Metabolic Imaging of Protein Synthesis in Bacteria by Widefield MIP

The incorporation of isotopes into cellular biomass leads to shifts in the IR absorption peaks to a lower wavenumber and has been observed for various isotopes and organisms.^32,55^ The effect of ^13^C incorporation by cells was previously demonstrated in a point-scan MIP system.^35,56,57^ Here, we use *E. coli* cells to demonstrate the capability of widefield MIP to image the metabolism of ^13^C from isotopically labeled compounds by bacteria. Glucose is a widely used energy source of bacteria, and various amino acids can be synthesized from glucose. Therefore, we selected ^13^C-labeled glucose as a model substrate for this study.

We imaged the cells under the MIP–FISH microscope (Figure 2). The rod shape of the individual *E. coli* was clearly shown in both the reflection and MIP images. We acquired multispectral widefield MIP images covering the protein amide I and amide II regions (1512 to 1768 cm^–1^) for the unlabeled glucose (^12^C-glucose) and ^13^C-labeled glucose (^13^C-glucose)-incubated cells (Figure S2). The ^12^C-glucose-incubated bacteria showed a protein amide I peak at around 1656 cm^–1^, while the protein amide I peak for ^13^C-glucose-incubated bacteria was around 1612 cm^–1^. The amide II peak also showed the isotopic effect with a smaller shift from 1548 to 1532 cm^–1^. The spectra from the cell-free background region showed no contrast, suggesting negligible effect from poly-l-lysine coating. We selected two key wavenumbers representing the ^12^C-protein (1656 cm^–1^, original amide I band) and ^13^C-protein (1612 cm^–1^, shifted amide I band) and recorded MIP images (Figure 2B,C,E,F). For the cells incubated with ^12^C-glucose, a higher intensity was observed at 1656 cm^–1^. For the cells incubated with ^13^C-glucose, a higher intensity was observed at 1612 cm^–1^ for the shifted amide I band, indicating the incorporation of the heavier carbon atoms into the protein. Due to the high throughput of widefield MIP, we were able to acquire high-SNR MIP images of up to hundreds of bacteria within 2.4 s.

To quantify the percentage of the ^13^C-protein in the whole protein pool, we defined a ^13^C-protein replacement ratio (Experimental Section and Figure S1). The estimated ^13^C-protein replacement ratio reaches 0.956 after 24 h based on the residual peak at 1656 cm^–1^ (Figure S2), which could be considered as near-full substitution.

We further demonstrate the high detection limit of isotope incorporation for MIP. We incubated *E. coli* cells for 24 h with varying percentages of ^13^C-glucose contributing to the total pool of available carbon. The ^13^C-protein replacement ratio was calculated for each group and plotted as the function of percentage of ^13^C-glucose in the medium (Figure 3). The ^13^C-protein replacement ratio increased as the percentage of ^13^C-glucose increased, and there was a clear linear correlation (*R*^2^ = 0.9982) between the concentration of ^13^C-glucose and the incorporation of ^13^C into the cellular protein. Notably, a significant difference was observed for 0 and 5% ^13^C-glucose incubation (pairwise *t*-test, *p* = 3.86 × 10^–22^). In comparison, with spontaneous Raman spectroscopy, a detection limit of 8% has been described for recording deuterium incorporation in microbial biomass.^21^ It should be noted that a relative low percentage of heavy water in Raman-based measurement is used to avoid potential inhibitory effects.^21^ Here, we observed no differences in growth or cell morphology that could reflect toxicity, even when all carbon source available was in the form of ^13^C-glucose (100% ^13^C-glucose). This is in agreement with literature reporting that incorporation of ^13^C-glucose shows negligible influence on cell metabolism and physiology.^58^

Heterogeneity in ^13^C incorporation was observed for the ^13^C-protein replacement ratio within each individual incubation group, despite the fact that cells were derived from an isogenic microbial population. To understand the origin of this heterogeneity, we performed multispectral MIP imaging on standard samples including polymer beads (PMMA beads 500 nm in diameter) and on a bovine serum albumin (BSA) film and calculated the mock ratio by applying a similar analysis as for the ^13^C-protein replacement ratio (Figure S4 and Table S4). The standard deviation for these mock ratios from the PMMA beads and BSA film is 5 times smaller than that of the *E. coli* samples, indicating that the ^13^C-protein replacement ratio fluctuation originated indeed mostly from phenotypic heterogeneity. This is not unexpected in batch incubations with the resulting physicochemical differences.

### Microbial Identification and Metabolism Analysis with MIP–FISH

To evaluate the capacity of MIP–FISH to simultaneously retrieve information on cellular metabolism and identity of the analyzed bacterial cells, we initially imaged *E. coli* cells that were stained by FISH with the oligonucleotide probe Gam42a–Cy5 (Figure 4 and Table S1). We acquired the FISH and MIP images sequentially with FISH imaging first to avoid photo bleaching. Hybridized cells showed a clear signal on the fluorescence Cy5 channel that overlaps well with the MIP images. As expected, in control experiments, no fluorescence signal could be detected in cells that were not hybridized. We observed for hybridized as well as nonhybridized cells higher IR intensities at 1612 cm^–1^ for cells grown in a ^13^C-glucose-containing medium (Figure 4). By calculating the ^13^C-protein replacement ratio, a difference in cells grown with unlabeled glucose and ^13^C-glucose was observed, as expected. However, the FISH process slightly reduced the ^13^C-protein replacement ratio (7.5% on average; Figure 4J). We also observed a higher than the 0 ^13^C-protein replacement ratio for ^12^C-glucose-incubated cells with FISH labeling (Figure S5). One potential reason can be that the selective loss of the cellular protein during the hybridization and washing steps of the FISH protocol leads to an overall amide I intensity decrease, changing the quantification coefficients. An effect of the FISH protocol on quantification of isotope incorporation within microbial cells has been previously reported for other vibrational spectroscopy-based methods.^20,21^ Therefore, we acquired coefficients for different cultures and treatment conditions to obtain a more accurate result (Table S2). Additionally, we observed no statistically significant difference between FISH probe Gam42a (specifically bind to *E. coli* rRNA) and FISH probe non-EUB (negative control, no binding), suggesting that the binding of rRNA-targeted probes will not influence our protein quantification.

We further tested the capacity of MIP–FISH to identify bacterial taxa and their metabolic status on multi-species samples. We started by using an artificial mixture of two common human gut microbiome members: *E. coli* and *B. thetaiotaomicron* (*B. theta*). *E. coli* cells grown in the presence of ^13^C-glucose were hybridized with the Gam42a–Cy5 probe, while *B. theta* cells were grown with ^12^C-glucose and hybridized with the Bac303–Cy3 probe (Table S1). Subtraction of MIP images at 1612 and 1656 cm^–1^ revealed that a fraction of the cells on this two-species sample displayed positive subtraction values (Figure 5B, yellow color), indicative of ^13^C-glucose incorporation, while the majority of the remaining cells displayed negative values (Figure 5B, blue color). From the subtraction results and the growth conditions, we inferred that cells with a positive contrast were *E. coli* and the cells with a negative contrast were *B. theta*. However, since both *E. coli* and *B. theta* are rod-shaped bacteria of similar size, we were not able to differentiate them based on morphology (Figure 5C). Benefiting from the fluorescence imaging capability of MIP–FISH and the ability of rRNA-targeted FISH to discriminate bacterial taxa, we could confirm that cells with a positive contrast were *E. coli* as these displayed a Cy5 fluorescence signal resulting from hybridization with the Gam42a–Cy5 probe (Figure 5A, red color). In contrast, *B. theta* cells displaying a Cy3 signal that originated from hybridization with the *Bacteroidales* probe Bac303–Cy3 (Figure 5A, green color) exhibited negative subtraction values. Finally, the ^13^C-protein replacement ratio was calculated (Figure 5D) and showed a significant difference between the two species (pairwise *t*-test, *p* = 9.74 × 10^–33^). A similar differentiation was observed for the opposite combination (*E. coli* incubated in ^12^C-glucose and FISH-labeled with Cy5, *B. theta* incubated in ^13^C-glucose and FISH-labeled with Cy3, Figure S6). Our results demonstrated that MIP–FISH is suitable to efficiently distinguish cells with ^13^C-induced protein peak shifts in mixed samples.

To test the performance of MIP–FISH beyond simple mixtures of bacteria and to demonstrate that it can be applied to identify microbes and retrieve metabolic information in a complex microbiome sample, we imaged a mixture of ^13^C-labeled *E. coli* and a human gut microbiome sample. The human large intestine is inhabited by trillions of gut microbes that perform a range of metabolic functions important for host health. We have therefore tested the suitability of MIP–FISH to investigate the microbial metabolism of isotopically labeled compounds in such a complex setting. MIP–FISH successfully enabled us to identify *E. coli* cells grown with ^13^C-glucose and hybridized with a Gam42a–Cy5 probe in a gut microbiome sample that had been incubated with ^12^C-glucose. The 1612 and 1656 cm^–1^ subtraction results (Figure 6B) and quantitative calculation of the ^13^C-protein replacement ratio (Figure 6D) together with fluorescence imaging showed the assimilation of ^13^C-glucose into protein for *E. coli* but not for other gut microbiome members (Figure 6A). As the gut microbiome contains many different microbial taxa, we measured a large number of cells (*n* = 593) to cover a majority of the species. From the replacement ratio analysis (Figure 6D), we observed a larger standard deviation when compared to pure bacteria sample; however, different gut microbiome member constitutions have no strong effect on differentiation between fully labeled and unlabeled cells. Together, these data show the applicability of MIP–FISH to a complex microbiome sample.

## Conclusions

In this study, we developed a MIP–FISH platform for in situ bacteria identification and metabolic imaging in a high-throughput manner with single-cell resolution. Benefiting from the high compatibility of MIP, we coupled a fluorescence module to a widefield MIP setup for fluorescence imaging of bacterial species hybridized with fluorescently labeled oligonucleotide probes. We demonstrated the potential for applying MIP–FISH on multi-species communities and complex samples and observed good correlations between MIP metabolic imaging and FISH imaging. As a proof of concept, we successfully applied MIP–FISH to image the microbial assimilation of ^13^C from labeled glucose. In pure culture experiments, high sensitivity was achieved with a detection limit of 5% of ^13^C in total carbon. In the complex microbiome sample containing many different unlabeled microbial taxa with different chemical cellular compositions, the background signal in the selected regions for the MIP-based detection of ^13^C-incorporation into proteins was higher, but unambiguous detection of spiked fully labeled *E. coli* cells was nevertheless possible. Microbial taxa with different physiologies and in distinct environments may display variable ^13^C incorporation levels and may never achieve full ^13^C-labeling. Under these circumstances, it would be important to first evaluate if MIP–FISH is able to unambiguously discriminate ^13^C-labeled and ^12^C-labeled cells of a taxa of interest in the context of a complex community.

Keeping in mind that the protein amide II band involves nitrogen, in future studies, MIP–FISH might also be suitable to study the assimilation of nitrogen-containing compounds using ^15^N-labeled substrates. More generally, this newly developed platform should now be ready to interrogate assimilation of many key substrates by human gut microbiome members including sweeteners, prebiotics, or even human-targeted drugs.

It is worth to compare MIP–FISH with other single-cell vibrational spectroscopy or IR-based metabolic characterization platforms for single-cell isotope probing of microbes. Single-cell spontaneous Raman spectroscopy offers a full-spectral coverage, but strong fluorescence background could complicate the spectral analysis.^21,31,59,60^ The throughput of Raman measurement is drastically improved by SRS at the cost of more expensive and complicated instrumentation, as well as limited spectral coverage.^20^ Additionally, it is harder to resolve ^13^C and ^15^N assimilation than ^2^H as the isotopic effect of ^13^C and ^15^N is relatively small and often buried in the Raman fingerprint region.^59^ On the other hand, IR offers a higher signal in the fingerprint region,^61^ which makes MIP a more suitable tool for high-throughput characterization of ^13^C and ^15^N assimilation in microbial cells. Additionally, widefield MIP provides a similar imaging speed to SRS, along with compactness, cost-effectiveness, and high compatibility with fluorescence merits.

We envision that the MIP–FISH platform will amend the toolbox of microbial ecologists and microbiome researchers that aim to simultaneously investigate the identity and function of individual microbial cells. Furthermore, this technique might also be useful for rapidly determining the antibiotic resistance of microbial cells in complex samples^62−67^ Finally, as a non-destructive single-cell analytic tool, it should be feasible in the future to integrate MIP imaging with other genotyping methods beyond FISH, such as cell-sorting and whole-genome sequencing.^68^

## Supporting Information Available

Data processing pipeline for metabolism quantification, multispectral widefield MIP imaging of unlabeled and ^13^C-labeled glucose-incubated *E. coli* cells, ultrafast MIP imaging 500 nm PMMA beads, multispectral imaging of the standard sample, influence of the FISH protocol on quantification, FISH probes used for single-cell analysis, reference coefficients for quantification, reference coefficients variation between measurements, mock ratio of standard samples, FISH procedure, and gut microbiome incubation (PDF)
