# Supplementary material for: Mid-Infrared Photothermal–Fluorescence In Situ Hybridization for Functional Analysis and Genetic Identification of Single Cells
Source: Anal Chem. 2023 Jan 18;95(4):2398–405. doi: 10.1021/acs.analchem.2c04474 (PMC9893215; doi:10.1021/acs.analchem.2c04474)
Supplement: Supplementary file 1 — ac2c04474_si_001.pdf [file ac2c04474_si_001.pdf]

Supporting Information for

Mid-Infrared Photothermal - Fluorescence in Situ Hybridization for Functional Analysis and Genetic Identification of Single Cells

Yeran Bai[1,3†], Zhongyue Guo[2,3†], Fátima C. Pereira[4], Michael Wagner[4,5*], Ji-Xin Cheng[1,2,3*]
[1]Department of Electrical and Computer Engineering, Boston University, Boston, MA 02215, USA

[2]Department of Biomedical Engineering, Boston University, Boston, MA 02215, USA

[3]Photonics Center, Boston University, Boston, MA 02215, USA
[3]Centre for Microbiology and Environmental Systems Science, Department of Microbiology and Ecosystem Science, University of Vienna, 1090 Vienna, Austria

[4]Department of Chemistry and Bioscience, Aalborg University, 9220 Aalborg, Denmark

† These authors contributed equally to this work.

*Corresponding authors. Emails: michael.wagner@univie.ac.at, jxcheng@bu.edu

This pdf file includes:
Figure S1. Data processing pipeline for high-throughput single-cell metabolic analysis from two IR wavenumber widefield MIP imaging.
Figure S2. Widefield MIP spectra of ^{12}C- and ^{13}C-glucose incubated *E. coli* cells.
Figure S3. Ultrafast imaging of 500 nm PMMA beads by the optimized widefield MIP setup.
Figure S4. Multispectral wide-field MIP imaging of standard samples and mock replacement ratio peaks.
Figure S5. Influence of FISH protocol on ^{13}C-protein replacement ratio quantification.
Figure S6. MIP-FISH imaging of an *E. coli* and *B. theta* mixture.
Table S1. FISH probe for single cell analysis.
Table S2. Reference coefficients used to calculate ^{13}C-protein replacement ratio and statistics on reported experiments.
Table S3. The coefficients obtained for the same reference sample on different days.
Table S4. Mock ratio of standard samples.
Supplementary Methods. Fluorescence *in-situ* hybridization. Gut microbiome incubation.

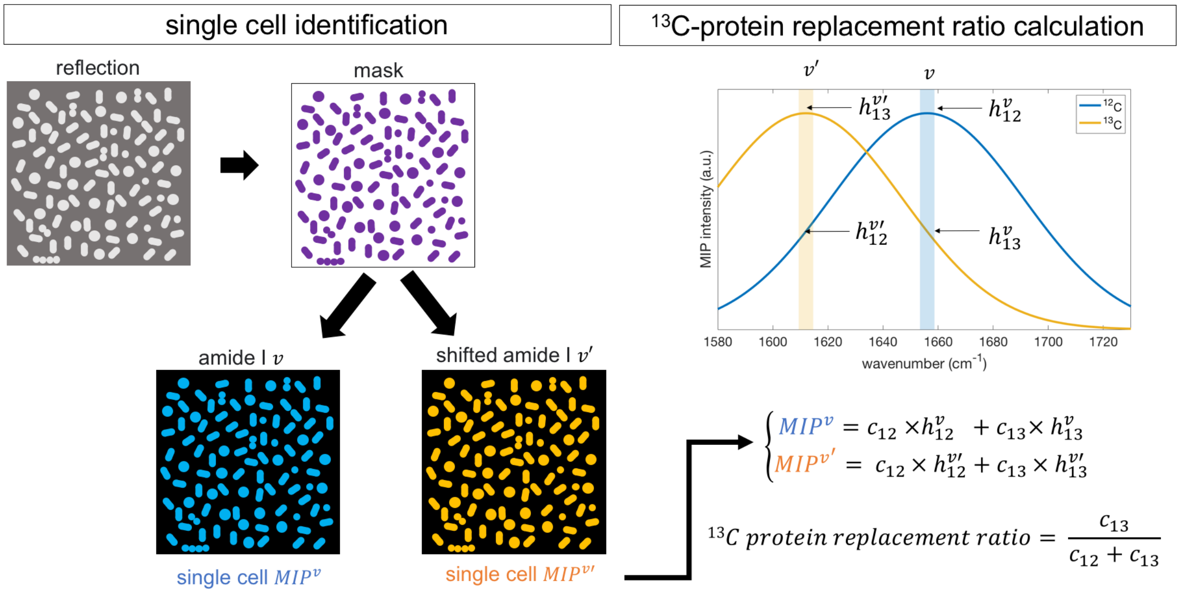

Figure S1. Data processing pipeline for high-throughput single-cell metabolic analysis from two IR wavenumber widefield MIP imaging. We imaged bacteria cells at two IR wavenumbers ν and ν' (center around 1656 and 1612 cm^{-1}) for MIP imaging. Individual cells were selected based on the reflection images and a corresponding mask was created. The mask was applied to MIP images to measure MIP intensities for each cell. A simulated protein amide I region for ^{12}C-glucose (blue curve) and ^{13}C-glucose (orange curve) incubated cells are shown on the right, with the ν and ν' indicated in blue and orange shaded lines. The measured MIP intensities at protein amide I band can be considered as a linear combination of ^{12}C-protein and ^{13}C-protein. We quantified the contribution from ^{12}C-protein and ^{13}C-protein (c_{12} and c_{13}) to the measured MIP intensities at ν and ν' ($MIP^{\nu}$and $MIP^{\nu'}$) based on the coefficients ($h_{12}^{\nu}, h_{12}^{\nu'}, h_{13}^{\nu}, h_{13}^{\nu'}$) obtained from the reference sample. The ^{13}C-protein replacement ratio is defined as $c_{13}/(c_{12} + c_{13})$. The coefficients used in the reported experiments are listed in Table S2.

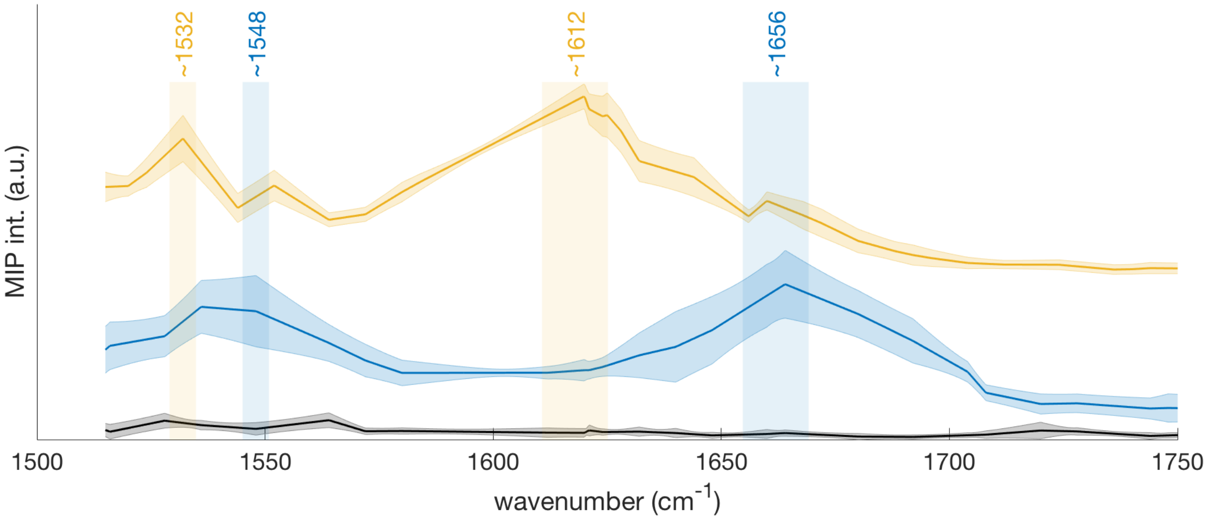

Figure S2. Widefield MIP spectra of ^{12}C- and ^{13}C-glucose incubated *E. coli* cells. The cells were incubated with ^{12}C-glucose (blue) or ^{13}C-glucose (orange) for 24 hours and cell-free region (black). MIP images were acquired by tuning the IR laser in the 1512 to 1768 cm^{-1} range with a step size around 10 cm^{-1}. Spectra were linear interpolated and offset to better visualize the trend. Standard deviation (shaded curve) of the spectra was determined from 10 single cells or background regions. The isotopic effect on protein spectra is clearly visualized by red-shifted peak (blue to orange line vertical bars). For ^{13}C-glucose incubated cells, a small residue peak can still be observed in the original peak positions for both the amide I and amide II bands. However, the area under this amide I residue peak is only 4.4% of the area of this broad peak in unlabeled *E. coli* cells, indicating a near-full substitution of ^{13}C in the total protein in *E. coli* cells incubated with ^{13}C-glucose for 24 hours.

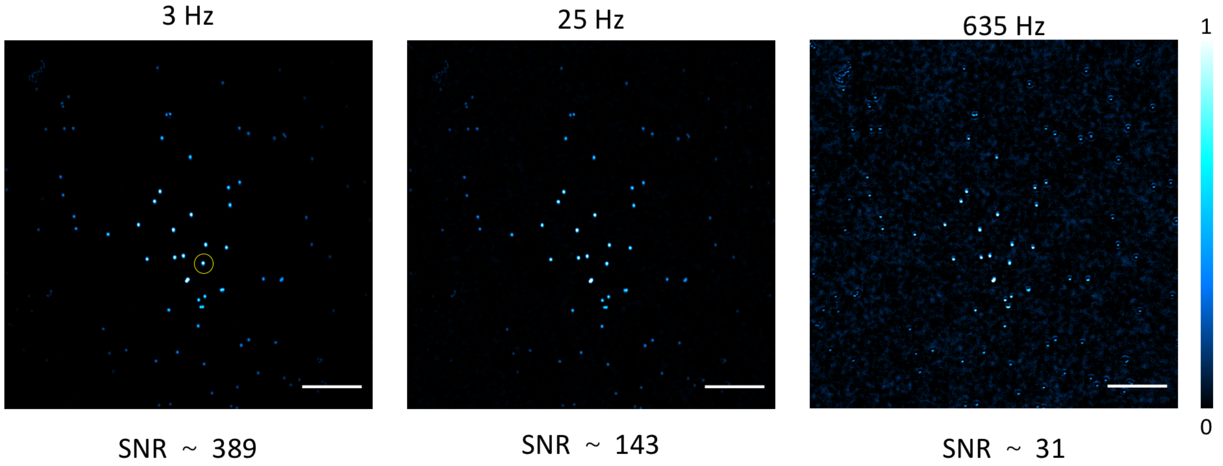

Figure S3. Ultrafast imaging of 500 nm PMMA beads by the optimized widefield MIP setup. PMMA beads were imaged at 1728 cm^{-1} at different speed through various averaging frames. The signal to noise ratio (SNR) was calculated as the maximum MIP intensity of a single bead (circled in 3Hz image) divided by the standard deviation of a surrounding background area. A reasonable SNR ~31 was achieved for ultrafast imaging at 635 Hz, which corresponds to single 'IR on' – 'IR off' frame subtraction. Scale bars: 10 µm.

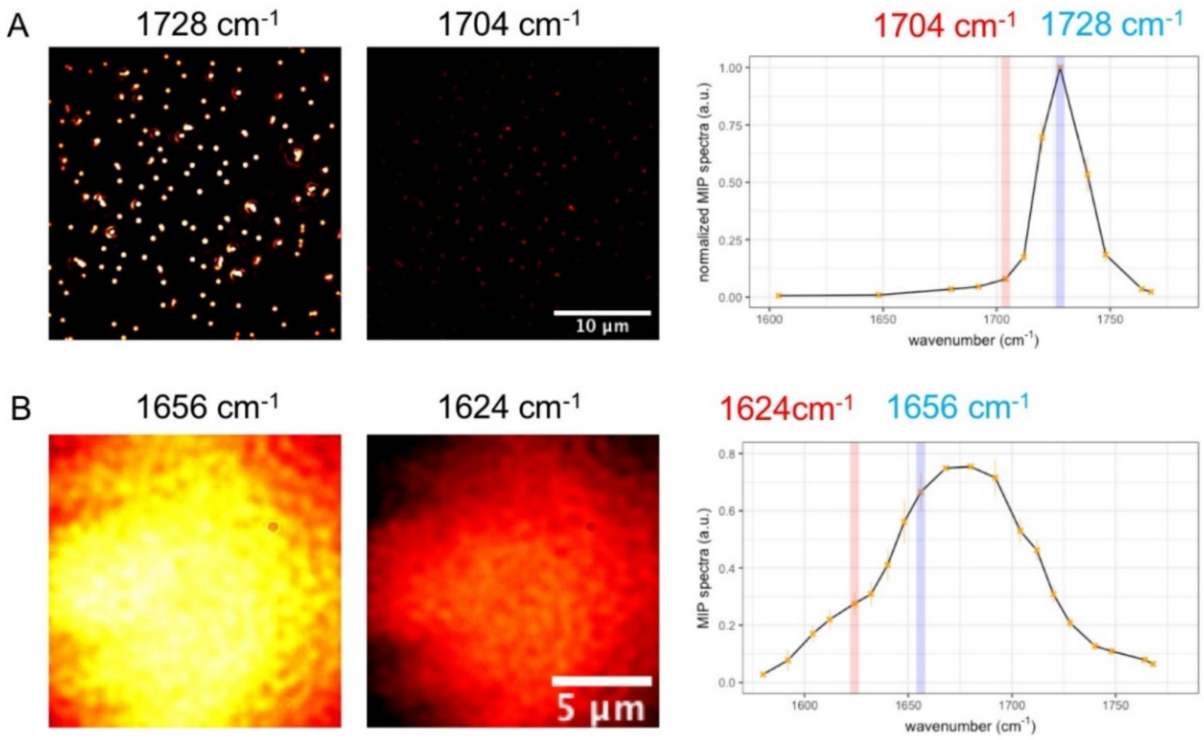

Figure S4. Multispectral wide-field MIP imaging of standard samples and mock replacement ratio peaks. For PMMA beads with 500 nm in diameter (A) and a bovine serum albumin (BSA) film (B), multispectral wide-field MIP images were acquired and the mock replacement ratio was calculated by applying an analogous analysis as for the ^{13}C-protein replacement ratio (see **Figure S1**). For PMMA beads, 1728 cm^{-1} and 1704 cm^{-1} was used as the mock original and shifted amide I peak. For the BSA film, 1656 cm^{-1} and 1624 cm^{-1} was used as the mock original and shifted amide I peak. Wide-field MIP images at selected mock peaks are shown along with the widefield spectrum.

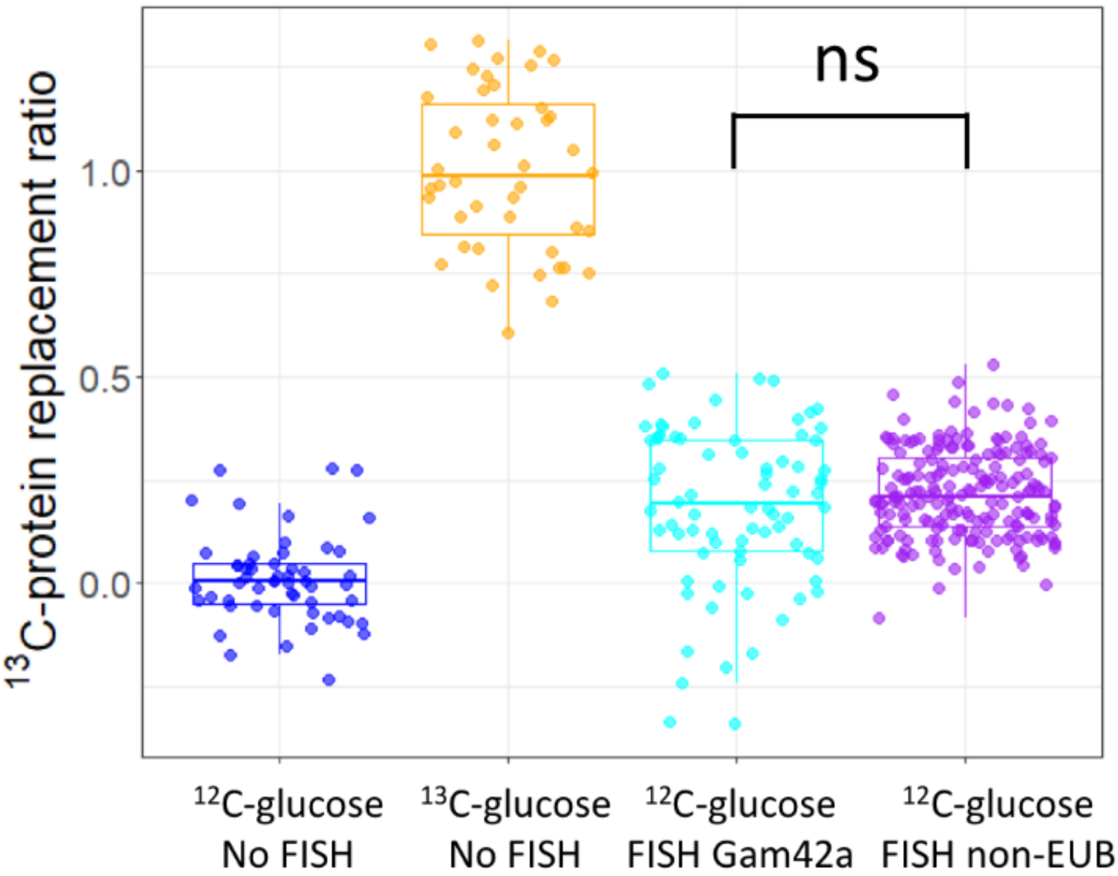

Figure S5. Influence of FISH protocol on ^{13}C-protein replacement ratio quantification. ^{12}C-glucose and ^{13}C-glucose incubated *E.coli* cells that were not FISH hybridized (No FISH) were used as references to infer the quantification coefficients (blue and yellow data points). ^{12}C-glucose incubated *E. coli* cells that were hybridized with the specific Gam42a FISH probe labelled with Cy5 or with a nonsense control FISH probe (non-EUB labelled with Cy5) showed a slight increase of the ^{13}C-protein replacement ratio compared with the corresponding *E. coli* cells that were not hybridized. The ^{13}C-protein replacement ratio inferred was not significantly different between Gam42a and non-EUB experiments (pairwise t-test, $p = 0.16$), suggesting that the hybridization or washing steps of the FISH protocol, but not the Cy5 fluorophore affect the quantification.

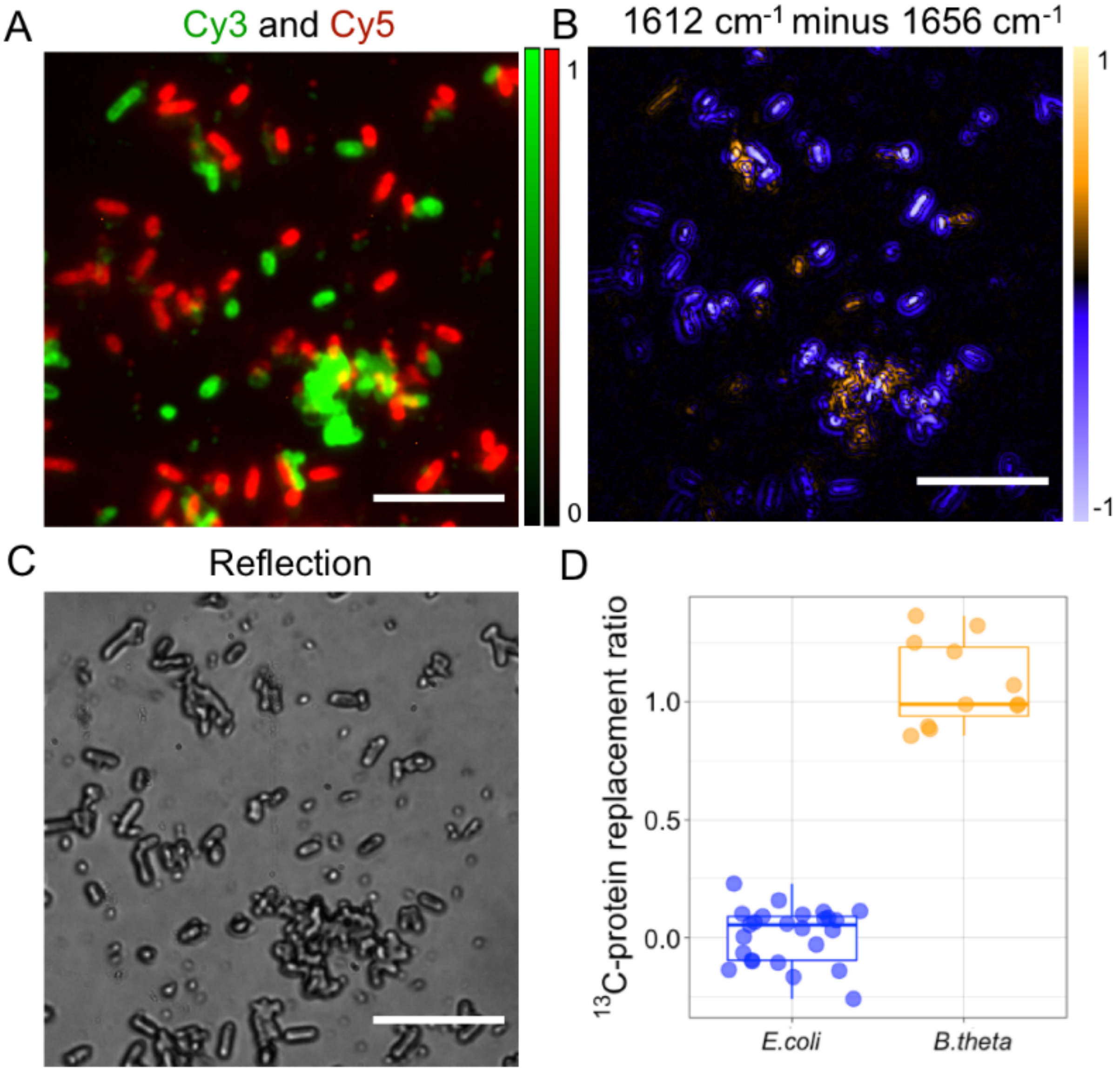

Figure S6. MIP-FISH imaging of an *E. coli* and *B. theta* mixture. In contrast to the experiment displayed in Figure 5, here *E. coli* cells were incubated with 0.4% (w/v) ^{12}C-glucose and hybridized with Gam42a-Cy5 oligonucleotide probe, while *B. theta* cells grown in the presence of 0.5% (w/v) ^{13}C-glucose were hybridized with a Bac303-Cy3 oligonucleotide probe. Subsequently, cells from both species were mixed and analyzed. (A) Fluorescence imaging for identification of *E. coli* (red) and *B. theta* (green). Scale bars 10 μm. (B) Subtraction of two MIP images (intensity at 1612 cm^{-1} minus intensity at 1656 cm^{-1}) showed that a portion of the cells have incorporated ^{13}C into the protein (in yellow) while other cells showed no ^{13}C labelling (in blue). (C) Reflection image shows cell morphology of the bacterial mixture. (D) Quantification of ^{13}C-protein replacement ratio. (Pairwise t-test: p=7.37e-11.)

Table S1. FISH probe for single cell analysis.

Probe Name	Sequence (5' to 3')	Formamide (%)	Major target taxa*	Reference
**Gam42a**	GCCTTCCCACTT CGTTT	35	Class Gammaproteobacteria (28%)	(Manz, Amann, Ludwig, Wagner, & Schleifer, 1992)
**Bac303**	CCAATGTGGGG GACCTT	10	Family Prevotellaceae (73%)<br>Family Barnesiellaceae (57%)<br>Family Tannerellaceae (50%)	(Manz, Amann, Ludwig, Vancanneyt, & Schleifer, 1996)
**NON-EUB**	ACTCCTACGGG AGGCAGC	-	negative control probe	(Wallner, Amann, & Beisker, 1993)

* According to Silva database v138.1 (https://www.arb-silva.de/). The probe coverage for each taxon is shown in parenthesis.

Table S2. Reference coefficients used to calculate ^{13}C-protein replacement ratio and statistics on reported experiments.

sample	Reference sample	Reference coefficients	Mean replacement ratio	standard deviation	number of ROIs
**Figure 3. High detection sensitivity for ^{13}C-incorporation.**					
0% ^{13}C-glucose	*E. coli* 0% ^{13}C-glucose<br>*E. coli* 100% ^{13}C-glucose	$h_{12}^{v} = 0.688$, $h_{12}^{v'} = 0.311$, $h_{13}^{v} = 0.289$, $h_{13}^{v'} = 0.711$	0.01100	0.07815	332
5% ^{13}C-glucose			0.06691	0.07627	454
10% ^{13}C-glucose			0.14437	0.06573	449
30% ^{13}C-glucose			0.35804	0.06516	155
50% ^{13}C-glucose			0.51838	0.12283	274
70% ^{13}C-glucose			0.69620	0.15375	243
90% ^{13}C-glucose			0.88839	0.11189	231
100% ^{13}C-glucose			0.99900	0.09107	212
**Figure 4. FISH is compatible with MIP metabolic imaging.**					
^{12}C-glucose, fixed cells, no FISH	*E. coli* ^{12}C-glucose no FISH<br>*E. coli* ^{13}C-glucose no FISH	$h_{12}^{v} = 0.626$, $h_{12}^{v'} = 0.374$, $h_{13}^{v} = 0.333$, $h_{13}^{v'} = 0.667$	0.01099	0.09424	115
^{13}C-glucose, fixed cells, no FISH			0.99900	0.12762	286
^{13}C-glucose, Cy5-labelled cells			0.92411	0.14720	553
**Figure 5. MIP-FISH imaging of bacterial mixtures.**					
E. coli (^{13}C, Cy5)	*E. coli* (^{12}C, Cy5)<br>*E. coli* (^{13}C, Cy5)	$h_{12}^{v} = 0.573$, $h_{12}^{v'} = 0.427$, $h_{13}^{v} = 0.298$, $h_{13}^{v'} = 0.702$	0.99764	0.20615	71

B. theta (^{12}C,Cy3)	*B. theta* (^{12}C,Cy3) *B. theta* (^{13}C,Cy3)	h_{12}^{v} = 0.618, $h_{12}^{v'}$ = 0.382, h_{13}^{v}= 0.444, $h_{13}^{v'}$ = 0.556	-0.00303	0.32610	53
**Figure 6. MIP-FISH imaging of a gut microbiome sample with spiked ^{13}C-labelled *E. coli* cells.**					
E. coli (^{13}C, Cy5)	*E. coli* 0% ^{13}C-glucose *E. coli* 100% ^{13}C-glucose	h_{12}^{v} = 0.674, $h_{12}^{v'}$ = 0.326, h_{13}^{v}= 0.276, $h_{13}^{v'}$ = 0.723	0.94167	0.07274	42
Gut microbiome			0.12506	0.16209	593
**Figure S5. Influence of FISH protocol on ^{13}C-protein replacement ratio measurements of ^{12}C-glucose incubated *E. coli* cells.**					
E. coli ^{12}C no FISH	*E. coli* ^{12}C no FISH *E. coli* ^{13}C no FISH	h_{12}^{v} = 0.674, $h_{12}^{v'}$ = 0.326, h_{13}^{v}= 0.392, $h_{13}^{v'}$ = 0.608	0.01100	0.10652	55
E. coli ^{13}C no FISH			0.99900	0.19486	44
E. coli ^{12}C FISH Gam42a			0.18481	0.19354	76
E. coli ^{12}C FISH non-EUB			0.21810	0.10642	191
**Figure S6. MIP-FISH imaging of an *E. coli* and *B. theta* mixture.**					
E. coli (^{12}C, Cy5)	*E. coli* (^{12}C, Cy5) *E. coli* (^{13}C, Cy5)	h_{12}^{v} = 0.573, $h_{12}^{v'}$ = 0.427, h_{13}^{v}= 0.298, $h_{13}^{v'}$ = 0.702	0.01268	0.11680	25
B. theta (^{13}C,Cy3)	*B. theta* (^{12}C,Cy3) *B. theta* (^{13}C,Cy3)	h_{12}^{v} = 0.618, $h_{12}^{v'}$ = 0.382, h_{13}^{v}= 0.444, $h_{13}^{v'}$ = 0.556	1.07379	0.18268	11

Table S3. The coefficients obtained for the same reference sample on different days. The reference sample here was the *E. coli* cells incubated with 0.2% (w/v) ^{12}C-glucose or ^{13}C-glucose for 24 hours and subsequently fixed but not hybridized, reported in Figure 3.

	**Day 1**	**Day 2**	**Day 3**
h_{12}^{v}	0.688	0.691	0.674
$h_{12}^{v'}$	0.311	0.309	0.326
h_{13}^{v}	0.289	0.298	0.276
$h_{13}^{v'}$	0.711	0.702	0.723

Table S4. Mock ratio of standard samples. The acquisition time was 0.31 second per IR wavenumber.

**Sample**	**Normal peak (cm-1)**	**Shifted peak (cm-1)**	**Mean replacement ratio**	**Standard deviation**	**Number of ROIs**
PMMA beads (500nm in diameter)	1728	1704	0.01299 (mock)	0.00895	40
BSA film	1656	1624	0.13313 (mock)	0.01553	72

Supplementary Methods

Fluorescence *in-situ* hybridization. Fixed cells (100 µL) were pelleted at 14,000 × *g* for 10 min, resuspended in 100 µL 96% analytical grade ethanol, and incubated for 1 min at room temperature for dehydration. Subsequently, the samples were centrifuged at 14,000 × *g* for 5 min, the ethanol was removed, and the cell pellet was air-dried. Cells were hybridized in solution (100 µL) for 3 h at 46 °C. The hybridization buffer consisted of 900 mM NaCl, 20 mM Tris-(hydroxymethyl)-amino methane HCl, 1 mM ethylenediamine tetraacetic acid, and 0.01% sodium dodecylsulphate and contained 100 ng of the respective fluorescently labelled oligonucleotide as well as the required formamide concentration to obtain stringent conditions (**Table S1**). After hybridization, samples were immediately transferred into a centrifuge with a rotor preheated at 46 °C and centrifuged at 14,000 × *g* for 15 min at the maximum allowed temperature (40 °C) to minimize unspecific probe binding. Samples were washed in a buffer of appropriate stringency (Daims, Stoecker & Wagner, 2006) for 15 min at 48 °C, and cells were centrifuged for 15 min at 14,000 × *g* at the maximum allowed temperature (40 °C) in a centrifuge with a rotor preheated at 46 °C. Cells were finally washed with 500 µL of ice-cold phosphate buffered saline, resuspended in 20 µL of phosphate buffered saline and stored at 4 °C until further use.

Gut microbiome incubation. Human fecal samples were collected from six healthy adult individuals (two male and four females between the ages of 26 to 39) who had not received antibiotics in the prior 3 months. Study participants provided informed consent and self-sampled using an adhesive paper-based feces catcher (FecesCatcher, Tag Hemi, Zeijen, NL) and a sterile polypropylene tube with the attached sampling spoon (Sarstedt, Nümbrecht, DE). The study protocol was approved by the University of Vienna Ethics Committee (reference No.00161). All meta(data) is 100% anonymized and compliant with the University's regulations. Samples were transferred into an anaerobic tent (Coy Laboratory Products, USA) within 30 min after sampling. Samples were suspended in M9 medium (prepared without glucose) to achieve a concentration of 0.1 g/ml, left to settle for 10 minutes, and the supernatants were combined. The combined fecal slurry was further diluted 10 times in this medium. The homogenate was left to settle for 10 minutes, and the supernatant was then distributed into glass vials. Each vial was supplemented with D-glucose (5 mg/ml; unlabelled D-Glucose, 99.5%, Sigma-Aldrich). After incubation for 6 h at 37 °C under anaerobic conditions (5% H_2, 10% CO_2, 85% N_2), sample aliquots were collected by centrifugation. Aliquots were fixed in 4% formaldehyde for 2 h at 4°C. Samples were finally washed two times with 1 ml of PBS and stored in PBS at 4 °C until further use.

Reference

Manz, W., Amann, R., Ludwig, W., Vancanneyt, M., & Schleifer, K.-H. (1996). Application of a suite of 16S rRNA-specific oligonucleotide probes designed to investigate bacteria of the phylum cytophaga-flavobacter-bacteroides in the natural environment. Microbiology, 142(5), 1097-1106.

Manz, W., Amann, R., Ludwig, W., Wagner, M., & Schleifer, K.-H. (1992). Phylogenetic oligodeoxynucleotide probes for the major subclasses of proteobacteria: problems and solutions. Systematic and applied microbiology, 15(4), 593-600.

Wallner, G., Amann, R., & Beisker, W. (1993). Optimizing fluorescent in situ hybridization with rRNA-targeted oligonucleotide probes for flow cytometric identification of microorganisms. Cytometry: The Journal of the International Society for Analytical Cytology, 14(2), 136-143.

Daims, H., Stoecker, K., and Wagner, M. (2006) Fluorescence in situ hybidisation for the detection of prokaryotes. In Molecular Micorbial Ecology. Osbourne, A.M., and Smith, C. (eds). BIOS Advanced Methods. New York, USA: Taylor and Francis, pp. 213–239.
